# Right ventricular stroke work index by echocardiography in adult patients with pulmonary arterial hypertension

**DOI:** 10.1186/s12872-021-02037-y

**Published:** 2021-04-30

**Authors:** Raluca Jumatate, Annika Ingvarsson, Gustav Jan Smith, Anders Roijer, Ellen Ostenfeld, Johan Waktare, Göran Rådegran, Carl Meurling, Anna Werther Evaldsson

**Affiliations:** 1 Department of Clinical Sciences Lund, Cardiology, The Echocardiographic Laboratory, The Section for Heart Failure and Valvular Disease, VO. Heart and Lung Medicine, Skåne University Hospital, Lund University, Skane University Hospital, Entrégatan 7, 221 85 Lund, Sweden; 2 Department of Clinical Sciences Lund, Clinical Physiology, Skane University Hospital, Lund University, Lund, Sweden; 3 Liverpool Heart and Chest Hospital, Liverpool, UK

**Keywords:** Echocardiography, Right ventricular stroke work index, Right heart catheterization

## Abstract

**Background:**

In adult patients with pulmonary arterial hypertension (PAH), right ventricular (RV) failure may worsen rapidly, resulting in a poor prognosis. In this population, non-invasive assessment of RV function is challenging. RV stroke work index (RVSWI) measured by right heart catheterization (RHC) represents a promising index for RV function. The aim of the present study was to comprehensively evaluate non-invasive measures to calculate RVSWI derived by echocardiography (RVSWI_ECHO_) using RHC (RVSWI_RHC_) as a reference in adult PAH patients.

**Methods:**

Retrospectively, 54 consecutive treatment naïve patients with PAH (65 ± 13 years, 36 women) were analyzed. Echocardiography and RHC were performed within a median of 1 day [IQR 0–1 days]. RVSWI_RHC_ was calculated as: (mean pulmonary arterial pressure (mPAP)—mean right atrial pressure (mRAP)) x stroke volume index (SVI)_RHC_. Four methods for RVSWI_ECHO_ were evaluated: RVSWI_ECHO-1_ = Tricuspid regurgitant maximum pressure gradient (TR_maxPG_) x SVI_ECHO_, RVSWI_ECHO-2_ = (TR_maxPG_-mRAP_ECHO_) x SVI_ECHO_, RVSWI_ECHO-3_ = TR mean gradient (TR_meanPG_) x SVI_ECHO_ and RVSWI_ECHO-4_ = (TR_meanPG_–mRAP_ECHO_) x SVI_ECHO_. Estimation of mRAP_ECHO_ was derived from inferior vena cava diameter.

**Results:**

RVSWI_RHC_ was 1132 ± 352 mmHg*mL*m^−2^. In comparison with RVSWI_RHC_ in absolute values, RVSWI_ECHO-1_ and RVSWI_ECHO-2_ was significantly higher (*p* < 0.001), whereas RVSWI_ECHO-4_ was lower (*p* < 0.001). No difference was shown for RVSWI_ECHO-3_ (*p* = 0.304). The strongest correlation, with RVSWI_RHC_, was demonstrated for RVSWI_ECHO-2_ (r = 0.78, *p* < 0.001) and RVSWI_ECHO-1_ ( r = 0.75, *p* < 0.001). RVSWI_ECHO-3_ and RVSWI_ECHO-4_ had moderate correlation (r = 0.66 and r = 0.69, *p* < 0.001 for all). A good agreement (ICC) was demonstrated for RVSWI_ECHO-3_ (ICC = 0.80, 95% CI 0.64–0.88, *p* < 0.001), a moderate for RVSWI_ECHO-4_ (ICC = 0.73_,_ 95% CI 0.27–0.87, *p* < 0.001) and RVSWI_ECHO-2_ (ICC = 0.55, 95% CI − 0.21–0.83, *p* < 0.001). A poor ICC was demonstrated for RVSWI_ECHO-1_ (ICC = 0.45, 95% CI − 0.18–0.77, *p* < 0.001). Agreement of absolute values for RVSWI_ECHO-1_ was − 772 ± 385 (− 50 ± 20%) mmHg*mL*m^−2^, RVSWI_ECHO-2_ − 600 ± 339 (-41 ± 20%) mmHg*mL*m^−2^, RVSWI_ECHO-3_ 42 ± 286 (5 ± 25%) mmHg*mL*m^−2^ and for RVSWI_ECHO-4_ 214 ± 273 (23 ± 27%) mmHg*mL*m^−2^.

**Conclusion:**

The correlation with RVSWI_RHC_ was moderate to strong for all echocardiographic measures, whereas only RVSWI_ECHO-3_ displayed high concordance of absolute values. The results, however, suggest that RVSWI_ECHO-1_ or RVSWI_ECHO-3_ could be the preferable echocardiographic methods. Prospective studies are warranted to evaluate the clinical utility of such measures in relation to treatment response, risk stratification and prognosis in patients with PAH.

## Background

In patients with pulmonary arterial hypertension (PAH), right ventricular (RV) function may deteriorate rapidly, constituting a negative prognostic factor for outcome [1, 2]. Non-invasive assessment of RV function is still challenging in patients with PAH and is mainly performed by transthoracic echocardiography. Compared to the left ventricle (LV), the morphology of the RV renders it more sensitive to changes in afterload (i.e. pressure overload) [3]. A simple surrogate measure of afterload by echocardiography is the calculation of systolic pulmonary arterial pressure (sPAP) by Bernoulli’s equation using the maximum pressure gradient from an existing tricuspid regurgitation together with estimated mRAP_ECHO_ [4, 5]. Clinically, RV afterload is generally defined as pulmonary vascular resistance (PVR) as measured by right heart catheterization (RHC). Using echocardiography, PVR can be assessed by the method suggested by Abbas et al. [6] or by using pulmonary acceleration time [4], however with poor accuracy. However, PVR does not quantify the compensation of RV work to changes in afterload [7]. With increasing PVR, the workload of the right ventricle will increase and vice versa. From a hemodynamical point of view, a more representative measure of the total RV workload is RV stroke work index (RVSWI), as it incorporates both stroke volume and pulmonary pressure, hence a determinant of RV failure [7]. Moreover, RVSWI_RHC_ also accounts for both the effect of preload, as it includes mean right atrial pressure (mRAP), as well as the effect of afterload, i.e. mean pulmonary pressure (mPAP). RVSWI being an invasive measure of RV function assessed by RHC, is calculated by the formula: RVSWI_RHC_ = (mPAP—mRAP) x stroke volume index (SVI).

In previous PAH studies, RVSWI_RHC_ has been shown to predict outcome in children [8] and adults [9, 10]. A proposed echocardiographic formula for RVSWI has been used in children with PAH. They calculated RVSWI by multiplying the tricuspid regurgitant maximum pressure gradient (TR_maxPG_) with RV stroke volume (SV) using the stroke volume from the RV outflow tract (RVOT) [7]. However, indexation to body surface area (BSA) was not incorporated, and estimation of mRAP was not considered in this formula. Theoretically, RVSWI can been calculated by echocardiography in several different ways using either TR_maxPG_ [7] or to mimic RHC, also incorporating mPAP. With echocardiography, mPAP can be estimated from the velocity integral of the TR doppler profile or from the early peak velocity of an existing pulmonary regurgitation with addition of mRAP_ECHO_ [4]. RVSWI derived from echocardiography (RVSWI_ECHO_) has, however, not been thoroughly compared to invasive measures in treatment naïve adults with PAH. Furthermore, the relevance of including BSA and mRAP in the echocardiographic equations are not elucidated. The aim of the present study was to evaluate the association and concordance between four different echocardiographically derived BSA-indexed methods of calculating RVSWI_ECHO_ to invasively calculated RVSWI_RHC_, with and without incorporating echocardiographically estimated mRAP.

## Methods

### Study population

Initially, seventy consecutive adult patients with treatment naïve PAH examined from January 1^st^ 2012 to December 31^th^ 2019 at Skane University Hospital, Lund were retrospectively evaluated. In accordance with contemporary guidelines [11], the diagnosis of PAH were made by RHC in absence of other causes of pre- and post-capillary pulmonary hypertension (PH). Pre-capillary pulmonary hypertension was defined according to at the time existing guidelines [11, 12] as mPAP ≥ 25 mmHg, pulmonary artery wedge pressure (PAWP) < 15 mmHg and PVR > 3 Wood Units (WU). The new suggested breakpoint of mPAP > 20 mmHg at WSPH 2018 in Nice [13] was adopted in the latter phase of the study. Medical records were used for retrieving patient characteristics. Inclusion criteria were maximally 7 days between echocardiography and RHC, provided no clinical deterioration nor change in medical treatment between the exams. No patients with surgical corrected or unrepaired shunts were included in the study. None of the patients had more than mild pulmonary- or mitral regurgitation and no patient had more than trivial aortic regurgitation. Tricuspid regurgitation was present as mild (61%) and moderate (39%). Exclusion criteria were atrial fibrillation (n = 2), poor echocardiographic image quality (n = 4), pacemaker (n = 1), non-adequate tricuspid spectral doppler (n = 1), cardiac by-pass surgery (n = 1), myocardial infarction with decreased left ventricular systolic function (n = 1), significant valvular disease (moderate aortic stenosis (n = 2), mitral stenosis (n = 1) and > moderate tricuspid regurgitation (n = 0)) and previous treatment for PAH (n = 3). After exclusions, 54 patients remained available for inclusion in the study.

### Echocardiography

All echocardiograms were obtained with an iE33 platform (Philips Healthcare, Eindhoven, NL) and a S5-1 transducer. Acquisition and assessment of echocardiographic measures were performed according to guidelines [14]. All echocardiographic images were stored digitally into an echocardiographic database (Philips IntelliSpace Cardiovascular, Philips Healthcare, Eindhoven, NL). Conventional RV function parameters as well as right ventricular free wall strain were assessed and analyzed as previously described by our research group according to guidelines [14, 15].

**Fig. 1:**
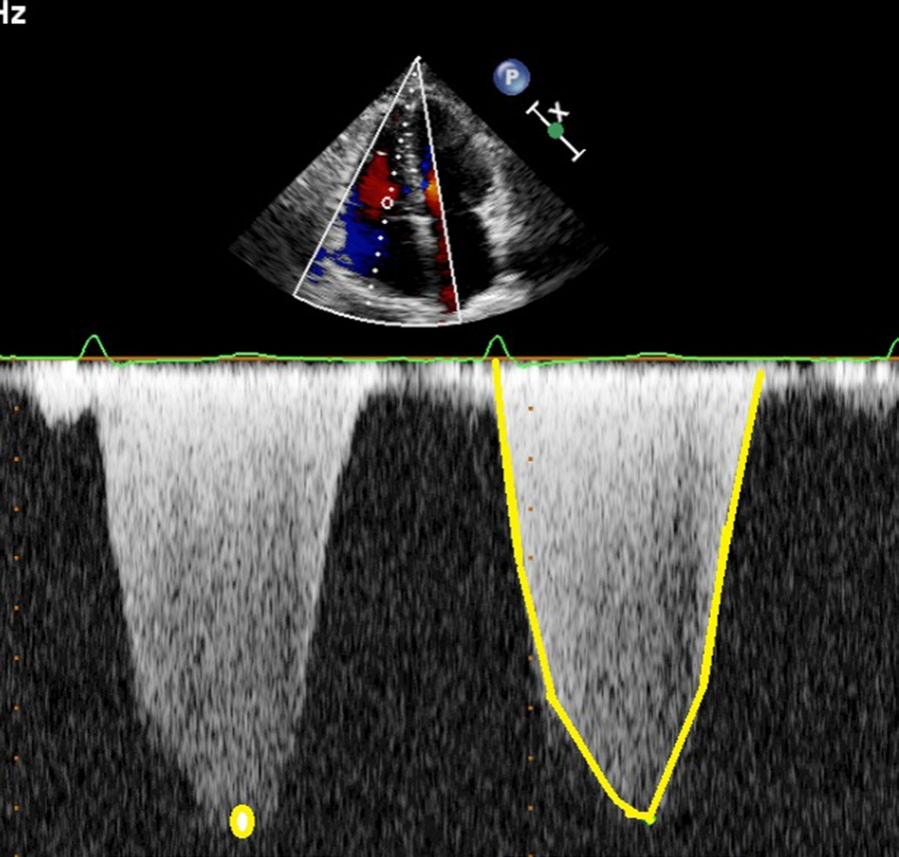
Echocardiographic illustration of measurement of maximum (yellow dot) and mean (yellow delineation) pressure gradients from a tricuspid regurgitation

The pressure gradient between RV and RA was assessed by the tricuspid regurgitant velocity (TR_maxPG_) using the modified Bernoulli Eq. (5), Fig. 1. The tricuspid mean pressure gradient (TR_meanPG_) was calculated by the velocity integral of the tricuspid regurgitant spectral doppler curve [4], Fig. 1. SVI_ECHO_ was calculated as: (left ventricular outflow tract (LVOT) area x velocity–time integral from LVOT)/BSA. For quantifying mRAP_ECHO_, the diameter of vena cava inferior and its collapsibility were used according to guidelines [14].

Four different echocardiographic methods for calculation of RVSWI_ECHO_ were evaluated:

1. RVSWI_ECHO-1_ = (TR_maxPG_) x SVI_ECHO_,
2. RVSWI_ECHO- 2_ = (TR_maxPG_—mRAP_ECHO_) x SVI_ECHO_,
3. RVSWI_ECHO- 3_ = TR_meanPG_ x SVI_ECHO_
4. RVSWI_ECHO-4_ = (TR_meanPG_—mRAP_ECHO_) x SVI_ECHO_.

### Right heart catheterization

The patients underwent RHC under local anesthesia in supine position at rest. An 8 French sheath was inserted using the Seldinger technique predominantly via the right internal jugular vein. A Swan-Ganz catheter was used for pulmonary arterial pressures measurements (sPAP, mPAP and diastolic PAP), together with mRAP and PAWP, recorded at free breathing over five heartbeats. Cardiac output (CO), (i.e. pulmonary blood flow in ml/min) was calculated by thermodilution in all patients. SV (ml/beat) was determined by dividing CO by heart rate (beats/min). SV was indexed to BSA to obtain SVI (stroke volume index). PVR was calculated using: (mPAP-PAWP)/CO. Systemic blood pressure was measured using an arm-cuff and sphygmomanometer. Mixed venous oxygen saturation was obtained for each patient. RVSWI_RHC_ was calculated as: (mPAP—mRAP) x SVI.

### Statistical analysis

Continuous data was expressed as mean ± standard deviation (SD) or median with inter-quartile range [IQR], as appropriate. Normality was assessed visually from histograms and confirmed by Kolmogorov Smirnov test. Categorical data was expressed in absolute numbers and proportion (percentage). The association between the four different measured RVSWI_ECHO1-4_ and the invasively measured RVSWI_RHC_ was evaluated using Pearson’s and intraclass correlation coefficients (ICC). The degree of correlation between tests was classified as either weak (r < 0.5), moderate (0.5–0.7), strong (0.7–0.9) or very strong (0.9–1.0) [16]. For calculating ICC, a two-way mixed model for absolute agreement between the measurements was used [17]. The 95% confidence interval (CI) of the ICC estimate was classified as either poor (< 0.5), moderate (0.5–0.75), good (0.75–0.9) and excellent reliability (> 0.9). Agreement of absolute measures from different modalities was computed as described by Bland and Altman [18]. Two-tailed P-values < 0.05 were considered statistically significant. Analyses were performed by commercially available software (IBM, SPSS Statistics, version 25, Chicago, IL, USA).

### Ethical aspects

The study complies with the Declaration of Helsinki and was approved by the regional department of the Swedish Ethical Review Authority (Dnr 2010/114, Dnr 2010/248 Dnr 2010/442). Written informed consent was given by the patients allowing analysis of all their clinical data including imaging, as granted in the ethical approval.

## Results

### Clinical characteristics

Demographics and baseline characteristics of the included 54 patients (36 women, 65 ± 13 years) are shown in Table 1. Patients underwent assessment with echocardiography and RHC within a median time of 1 day [IQR 0–1 days] and were all diagnosed with PAH. The causes of PAH can be found in Table 1. No PAH disease specific treatment was given before the exams (i.e. all patients were PAH treatment naïve). Most of the patients were in WHO-functional class II and class III (30% and 61%, respectively).

**Table 1:** Baseline characteristics for demographic, clinical and laboratory parameters

Number of patients (n)	54
Sex (women/men)	36/18
Age (years)	65 ± 13
BSA (m^2^)	1.8 ± 0.2
Etiological subclasses of pulmonary arterial hypertension	
Idiopathic PAH	30 (56)
Heritable PAH	3 (5)
PAH associated with CTD	17 (31)
PAH associated with portal hypertension	2 (4)
Drug and toxin induced PAH	2 (4)
Laboratory parameters	
NT-proBNP (ng/L)	1698 [375–3147]
Hemoglobin (g/L)	141 ± 19
Creatinine (µmol/L)	94 ± 30
Comorbidities	
Diabetes	15 (28)
Hypertension	24 (44)
Coronary artery disease	10 (19)
Previous stroke	3 (5)
Thyroid disease	14 (26)
Functional class, NYHA	
I	1 (2)
II	16 (30)
III	33 (61)
IV	4 (7)
Medication	
O_2_	12 (22)
Diuretics	26 (48)
Calcium antagonists	12 (22)
Anticoagulation	7 (13)

Data are expressed as mean ± SD, median [inter-quartile range] or as number. Categorical data is2) expressed in absolute numbers and proportion (percentage). BSA (body surface area), CTD (connective tissue disease), NT-proBNP (brain natriuretic peptide), NYHA class (New York Heart Association) functional classification for heart failure

Baseline echocardiographic and RHC characteristics are shown in Table 2. All patients exhibited normal LV size and normal LV ejection fraction. The RV was dilated and had an impaired function measured by RV fractional area change and free wall strain compared to reference values [14]. The left atrium was within normal range whereas the right atrium was enlarged [14]. Median severity of the tricuspid regurgitation was mild. The pressures obtained by echocardiography were 69 ± 17 mmHg for TR_maxPG_, 39 ± 9 mmHg for TR_meanPG_ and mean mRAP_ECHO_ was 8 [IQR 3–8] mmHg. By RHC, the patients exhibited mild to moderately raised mPAP (47 ± 12 mmHg), normal PAWP (7 [IQR 5–10] mmHg), a moderate-severely elevated PVR (10 ± 5 WU), a normal CO (4.2 ± 1.2 L/min) and a normal to slightly elevated mRAP_RHC_ of 7 ± 5 mmHg.

**Table 2:** Echocardiographic and right heart catheterization characteristics

BSA (m^2^)	1.8 ± 0.2
HR echo (beats/min)	83 ± 15
Echocardiographic characteristics	
IVSd (mm)	10.3 ± 0.2
LVIDd (mm)	40.9 ± 7.5
LVPWd (mm)	9.3 ± 2.1
LVEDV (mL)	57 ± 23
LVESV (ml)	23 ± 14
LVEF (%)	61 ± 10
LA Volume/BSA (mL/m^2^)	23 ± 11
RA volume/BSA (mL/m^2^)	43 ± 20
RA area (cm^2^)	22 ± 7
RVDd (mm)	36 ± 6
RV size inflow (mm)	48 ± 10
RV size mid cavity (mm)	37 ± 9
RVFAC (%)	27 ± 12
TAPSE (mm)	17 ± 5
s´ (cm/sec)	10 ± 2.9
RVFWS (%)	− 13.4 ± 4.7
SVI (ml/m^2^)	28.4 ± 8.7
CO (L/min)	4.2 ± 1.3
CI (L/min/m^2^)	2.3 ± 0.6
TR Vmax (m/sec)	4.1 ± 0.5
TR maximum gradient (mmHg)	69 ± 17
TR mean gradient (mmHg)	39 ± 9
mRAP (mmHg)	8 [3–8]
IVCd (mm)	19 ± 5
Right heart catheterization characteristics	
SPAP (mmHg)	76 ± 19
DPAP (mmHg)	29 ± 11
mPAP (mmHg)	47 ± 12
PAWP (mmHg)	7 [5–10]
mRAP (mmHg)	7 ± 5
SVI (ml/m^2^)	29.4 ± 8.4
CO (L/min)	4.2 ± 1.2
CI (L/min/m^2^)	2.3 ± 0.6
PVR (WU)	10.3 ± 5.0
SAP (mmHg)	138 ± 21

Data are expressed as mean ± SD or median [inter-quartile range]. BSA (body surface area), HR (heart rate), IVSd (intra ventricular septum diameter), LV (left ventricle), LVIDd (LV inner diastolic diameter), LVPWd (LV posterior wall diameter), LVEDV (LV end-diastolic volume), LVESV (LV end-systolic volume), LVEF (left ventricular ejection fraction), LA (left atrium), RA (right atrium), RV (right ventricle), RVDd (RV diastolic diameter in parasternal long axis view), RVFAC (RV fractional area change), TAPSE (tricuspid annular plane systolic excursion), S´ (peak systolic velocity of the lateral tricuspid valve annulus), RVFWS (right ventricular free wall strain), SVI (stroke volume index), CO (cardiac output), CI (cardiac index), TR (tricuspid regurgitation), TR Vmax (TR maximum velocity), mRAP (mean right atrial pressure), IVCd (inferior vena cava diameter), SPAP (systolic pulmonary arterial pressure), DPAP (diastolic pulmonary arterial pressure), mPAP (mean pulmonary arterial pressure), PAWP (pulmonary arterial wedge pressure), WU (Wood Units), PVR (pulmonary vascular resistance), SAP (systolic systemic arterial pressure)

### Correlation between echocardiographic measures and RHC

**Fig. 2:**
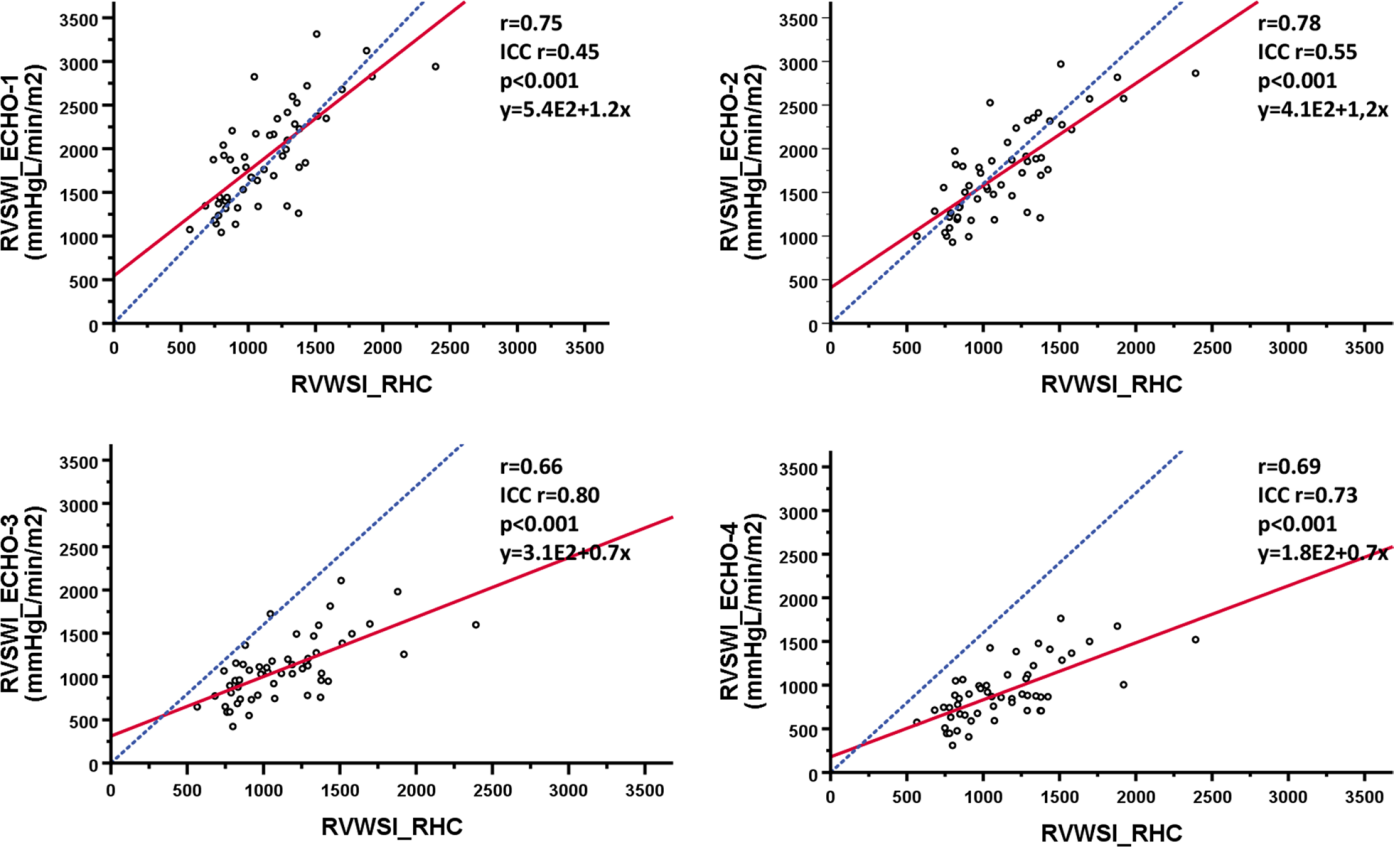
Scatterplots with regression lines in red, delineates the correlation between right ventricular stroke work index (RVSWI) derived from echocardiography RVSWI_ECHO1-4_ and right heart catheterization (RHC). The blue line represents the reference line. Pearson’s correlation coefficients (r-values) and intra class correlation (ICC) are depicted in the figure. E2 means equal to 100

**Fig. 3:**
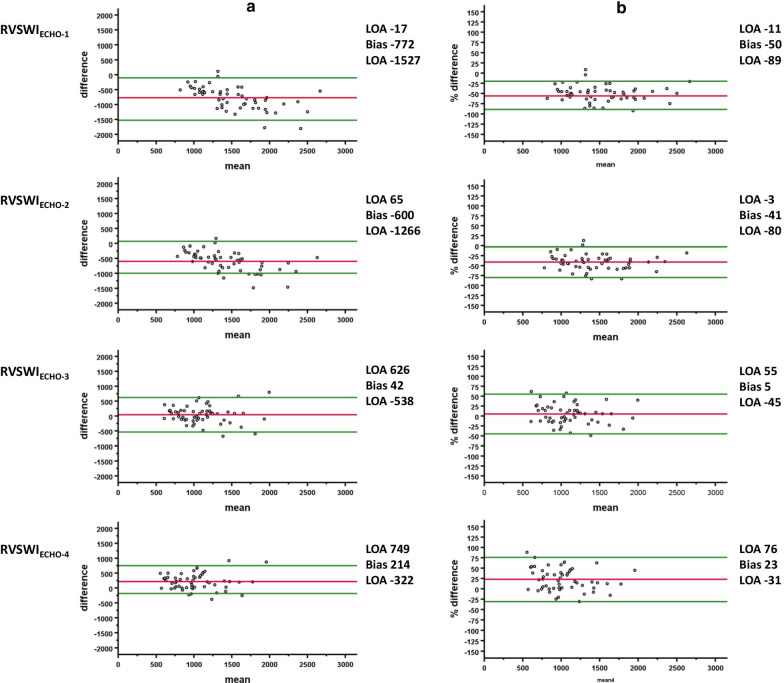
**a** Bland–Altman plots illustrating the agreement of right ventricular stroke work index (RVSWI) by right heart catheterization and the four different echocardiographic methods for calculation of RVSWI (RVSWI_ECHO1-4_). The red lines represent the absolute bias and the green lines represents the level of agreement (LOA). **b** Bland–Altman plots based on the percentage differences in RVSWI between right heart catheterization and echocardiography. The red lines represent the relative bias and the green lines represents the level of agreement (LOA)

RVSWI derived from echocardiography and RHC are shown in Table 3. In comparison with RVSWI_RHC_, RVSWI_ECHO-1_ and RVSWI_ECHO-2_ were significantly higher (*p* < 0.001) whereas RVSWI_ECHO-4_ were significantly lower in absolute values (*p* < 0.001). There was no difference for RVSWI_ECHO-3_ (*p* = 0.304). A strong correlation with RVSWI_RHC_ was demonstrated for RVSWI_ECHO-2_ and RVSWI_ECHO-1_ (r = 0.78 and r = 0.75, *p* < 0.001) followed by a moderate correlation for RVSWI_ECHO-3_ and RVSWI_ECHO-4_ (r = 0.66 and r = 0.69, *p* < 0.001 for all) as illustrated in Fig. 2. A good agreement (ICC) was demonstrated for RVSWI_ECHO-3_ (ICC = 0.80, 95% CI 0.64–0.88, *p* < 0.001), a moderate for RVSWI_ECHO-4_ (ICC = 0.73_,_ 95% CI 0.27–0.87, *p* < 0.001) and RVSWI_ECHO-2_ (ICC = 0.55, 95% CI -0.21–0.83, *p* < 0.001). A poor ICC was demonstrated for RVSWI_ECHO-1_ (ICC = 0.45, 95% CI -0.18–0.77, *p* < 0.001). The biases between the methods are illustrated in Fig. 3 and in Table 3, demonstrating the lowest bias for the calculated method RVSWI_ECHO-3_.

**Table 3:** Right ventricular stroke work index with right heart catheterization and echocardiography. Demonstrating differences in absolute values as well as biases (absolute and relative)

RVSWI (mmHg x mL/m^2^)	mean ± SD	Absolute bias	Relative bias (%)
RVSWI_RHC_	1132 ± 352		
RVSWI_ECHO-1_	1904 ± 568^***^	− 772 ± 385	− 50 ± 20
RVSWI_ECHO-2_	1732 ± 531^***^	− 600 ± 339	− 41 ± 20
RVSWI_ECHO -3_	1090 ± 366 ^#^	42 ± 286	5 ± 25
RVSWI_ECHO-4_	918 ± 336 ^***^	214 ± 273	23 ± 27

Data are expressed as means ± SD or as percentage. ***p < 0.001, ^#^*p* = 0.304. RVSWI (right ventricular stroke work index), RHC (right heart catheterization), ECHO (echocardiography)

## Discussion

To our knowledge this is the first study comparing RVSWI_RHC_ with four different echocardiographic derived methods for calculation of RVSWI in treatment naïve adult patients with PAH. Our results show a small bias and no significant difference in absolute values between RVSWI_RHC_ and RVSWI_ECHO-3_ (using the mean tricuspid gradient and SVI_ECHO_), albeit a moderate correlation between the methods. For the other three echocardiographic measurements, moderate to strong correlations with RVSWI_RHC_ were demonstrated, but poor concordance between values owing to larger biases.

### The concept of RVSWI

RVSWI is the product of measured mRAP, mPAP and SV derived from RHC. Consequently, RVWSI incorporates elements of RV preload, afterload as well as contractile performance [8]. Previous studies have suggested that RVSWI could be used as a marker of clinical outcome since it reflects total RV workload [19, 20]. In patients who have undergone lung transplantation, a higher RVSWI predicted worse outcomes [19]. RVSWI has also been used in patients with severe left ventricular heart failure eligible for left ventricular assist device to determine if the RV is sufficiently strong to meet the demands of the left ventricle and if a supplementary RV assist-device is necessary [20]. In this setting a cut-off value for RVSWI ≤ 250 mmHg x mL/m^2^ is associated with a need of RV assist-device.

The role of RVSWI in patients with PAH is still an unexplored field. In children as well as in adults with PAH, RVSWI has, however, shown a prognostic value [8–10] but data are sparse.

The interpretation of RVSWI is however complicated and general cut off values for RVSWI has not been established. Initially, a high RVSWI indicates an increased workload of the RV, required as it strives to overcome an increased pulmonary vascular stiffness [21]. Clinically this is demonstrated in the early stages of PAH, whereas the RV can adapt physiologically with hypertrophy and increased contractility but without significant changes in size [22]. In the progress of the disease, RV dilatation occurs as a compensatory mechanism to maintain an adequate SV [22]. Theoretically, in this stage RVSWI reaches its plateau phase. Eventually, with a continuous rise in PVR, these compensatory mechanisms are inadequate resulting in progressive impairment in RV contractility and consequently a decrease in both SV and mPAP [22]. In this scenario RVSWI decreases. However, in clinical practice, specific drug therapy is initiated in the majority of patients diagnosed with PAH [11]. In this scenario, if the patient responds to treatment, RVSWI is affected due to a decrease in mPAP and an increase of SV. Nevertheless, since no reference values has been established, comparison of absolute RVSWI values between patients is challenging. Therefore, in clinical practice, the role of RVSWI may be best suited in follow-up assessments where the patients are their own reference. Even if the clinical use of RVSWI in this setting seems to be a promising tool it requires a RHC.

### Echocardiographic calculated RVSWI

Finding new ways to noninvasively assess RV function in patients with PAH is of great importance. Although echocardiographic assessment of the RV has several limitations owing to its complex geometry, which challenges clinicians in daily practice, it is still the preferred imaging modality in daily routine. In this study we examined four different methods for non-invasive calculations of RVSWI using echocardiography-derived SV, pulmonary pressures and mRAP.

SV may be derived from either RVOT, or from LVOT. Unless there is intracardiac shunting or regurgitation of one or both semilunar valves present the SV may be considered equal. Since SV calculated from RVOT has several pitfalls, especially measuring the RVOT diameter [23], we used measurements from LVOT. In our study none of the patients had intra cardiac shunts or an aortic- or pulmonary regurgitation. Consequently, SV calculated from LVOT was considered equal to SV calculated from RVOT in this study.

Using echocardiography, calculation of systolic and mean pulmonary arterial pressure can be estimated from the spectral doppler of an existing tricuspid regurgitation. In two of the methods (RVSWI_ECHO-1_ and RVSWI_ECHO-2_), the TR_maxPG_ was used since it is an established non-invasive method for estimation of systolic pulmonary arterial pressures. The advantage using TR_maxPG_ compared to the mean gradient is the accessibility since it is present in most PAH patients and easy to obtain. By echocardiography mPAP can be assessed in several ways [4]. In this study the velocity integral was used in two of the methods (RVSWI_ECHO-3_ and RVSWI_ECHO-4_) since it has proven to exhibit a value closer to mPAP by RHC than the other methods [24, 25]. For the echocardiographic measurements using TR_maxPG_ (RVSWI_ECHO-1_ and RVSWI_ECHO-2_) a strong correlation to RVSWI_RHC_ were demonstrated, but poor agreement to the absolute values with large biases to be addressed. Incorporation of mRAP (RVSWI_ECHO-2_) did not change the results. This is in alignment with a study in children with PAH, where mRAP were not included, and a similar correlation was demonstrated [7].

For the echocardiographic measurement using TR_meanPG_, (RVSWI_ECHO-3_ and RVSWI_ECHO-4_, respectively) a moderate correlation to RVSWI_RHC_ were demonstrated. The method using only mPAP (RVSWI_ECHO-3_) showed no difference in absolute values compared to RVSWI_RHC_. Therefore, incorporation of echocardiographic estimated mRAP using IVC had no incremental value in non-invasive calculation of RVSWI_ECHO_, probably due to its poor accuracy in the clinical settings [26]. In comparison to RHC, none of the echocardiographically derived methods could demonstrate both good correlation and high agreement in absolute values. The differences between invasively and echocardiographic derived RVSWI could also be explained by errors from both modalities.

By echocardiography, potential sources or error consist of inaccuracy in calculating SV as well as the pulmonary pressures. Concerning SV, measuring the diameter from an outflow tract has well known limitations. Calculations of LVOT-flow and TR-gradient are Doppler-derived. Thus, they both are angle dependent which can lead to underestimation. Moreover, overestimation must be avoided by ensuring that measurements are performed only on well-defined spectral curve. Another source of bias in measurements of pulmonary pressures between the methods is estimation of mRAP by echocardiography which consequently could lead to disagreement. Concerning RHC, thermodilution can overestimate SV, especially in low cardiac output states. Moreover, pressure calculations by RHC are dependent on correct calibration and could be affected by respiration and arrhythmias.

However, the results suggest that either RVSWI_ECHO-1_ could be the preferred method according to its level of correlation or RVSWI_ECHO-3_ according to its better agreement with RHC. One major drawback of RVSWI is to identify if a decrease in RVSWI is due to treatment response (lower mPAP) or RV failure since both results in a decrease in TR pressure gradient. Additional clinical parameters, such as 6-min walk test, NT-proBNP, echocardiographic RV function parameters or changes in SVI, could be considered for differentiation.

### Clinical implications

In our PAH-clinic, we routinely perform serial echocardiographic evaluations, right heart catheterizations and clinical assessments in combination with 6-min walk tests according to ESC:RRS risk stratification [11]. Even if conventional echocardiographic parameters (i.e. TAPSE, RVFAC and RVFWS) have an established role in the clinical assessment of RV function, they have not been implemented in the risk assessment algorithm in PAH [11]. In the clinical setting it is of great importance to early identify a failing RV in order to in a timely manner determine when the patient needs to be listed for lung transplantation. Consequently, an exploration to identify useful echocardiography-derived measurements besides RA area and the presence of pericardial effusion are warranted. RVSWI measured by echocardiography could be a new method for prognosis assessment in adult patients with PAH preferable for follow-up in selected cases where the patients are their own reference. However, it is highly unclear at present whether echocardiographically derived RVSWI have additive value in these patients. Certainly, more studies are needed to understand the clinical implication on how to interpret RVSWI_ECHO_ and its role in management of these patients.

### Limitations

There are some limitations to our study. The use of invasively measured RVSWI for estimation of prognosis in PAH patients is not fully explored, which makes the utility of echocardiographically RVSWI also unclear. Consequently, the results of this study should merely be described as a proof of concept study.

The number of patients is furthermore relatively small. However, PAH is a rare disease and it is therefore difficult to obtain large study cohorts. Echocardiography and RHC were not performed simultaneously. However, most patients had examinations the same day or the following day with no medical changes. Even though the delay was minimal, this could have influenced the result.

Systemic blood pressure was only obtained invasively and not in conjunction to the echocardiographic examination that could to some extent have affected the results.

A concern is that our study estimated CO from the LVOT considering it equal to CO from the RVOT. However, in the presence of a severe tricuspid regurgitation, the RV-workload could increase. Thus, RVSWI would be inaccurate. Our study population comprised patients with mainly mild tricuspid regurgitation and none with more than moderate TR. Moreover, SVI calculated by echocardiography and by RHC were similar. This is despite the fact that moderate or severe tricuspid regurgitation is associated with underestimation of cardiac output measured by thermodilution [27], further exacerbating any difference. We therefore conclude that this issue did not affect the present study, but feel that the validity of derived calculation will lose accuracy and validity in the presence of severe TR.

By echocardiography, CO derived from RVOT measurement is challenging in most patients and have well known limitations in clinical practice [28].

## Conclusion

The correlation between RVSWI_RHC_ and all echocardiographic calculations of RWSVI was moderate to strong, while only RVSWI_ECHO-3_ displayed high concordance of absolute values. Incorporation of echocardiography-estimated mRAP had no incremental value in non-invasive calculation of RVSWI. In this study we were unable to find any single method that showed both good correlation and high agreement in absolute values compared to RHC. However, the results suggest that either RVSWI_ECHO-1_ could be the preferred method according to its level of correlation or RVSWI_ECHO-3_ according to its agreement to RHC. Future studies are needed to establish cut-off value for echocardiographically measured RVSWI and to evaluate the clinical utility of such measures in relation to PAH treatment response, risk stratification and prognosis.

## Data Availability

The datasets generated and/or analysed during this study are not available for publications due to research subject confidentiality. They are available in a unidentified form from the corresponding author on reasonable request.

## Abbreviations

BSA: Body surface area
CO: Cardiac output
ECHO: Echocardiography
ICC: Intraclass correlation coefficients
LV: Left ventricle
LVOT: Left ventricular outflow tract
mPAP: Mean Pulmonary arterial pressure
mRAP: Mean right atrial pressure
PAH: Pulmonary arterial hypertension
PAP: Pulmonary arterial pressure
PH: Pulmonary hypertension
PVR: Pulmonary vascular resistance
RA: Right atrium
RHC: Right heart catheterization
RV: Right ventricle
RVOT: Right ventricular outflow tract
RVFAC: Right ventricular fractional area change
RVFWS: Right ventricular free wall strain
RVSWI: Right ventricular stroke work index
SV: Stroke volume
SVI: Stroke volume index
TAPSE: Tricuspid annular plane systolic excursion
TR_maxPG_: Tricuspid regurgitation maximum pressure gradient
TR_meanPG_: Tricuspid mean pressure gradient
